# Effect of mechanistic target of rapamycin inhibitors on postrenal transplantation malignancy: A nationwide cohort study

**DOI:** 10.1002/cam4.1676

**Published:** 2018-08-16

**Authors:** Yi‐Chou Hou, Yen‐Chen Chang, Hao‐Lun Luo, Kuo‐Cheng Lu, Po‐Huang Chiang

**Affiliations:** ^1^ Division of Nephrology Department of Internal Medicine Cardinal Tien Hospital School of Medicine Fu‐Jen Catholic University New Taipei City Taiwan; ^2^ Graduate Institute of Clinical Medicine College of Medicine Taipei Medical University Taipei Taiwan; ^3^ Institute of Population Health Sciences National Health Research Institutes Zhunan Taiwan; ^4^ Graduate Institute of Medicine College of Medicine Kaohsiung Medical University Kaohsiung Taiwan; ^5^ Department of Urology Kaohsiung Chang Gung Memorial Hospital Chang Gung University College of Medicine Kaohsiung Taiwan; ^6^ Division of Nephrology Department of Medicine Tri‐Service General Hospital National Defense Medical Center Taipei Taiwan

**Keywords:** malignancy, mechanistic target of rapamycin inhibitor, renal transplantation

## Abstract

**Background:**

Post‐transplantation malignancy influenced graft survival and overall survival in the patients receiving renal transplantation. Immunosuppressants influenced the immune surveillance, but whether immunosuppressive agents have impact for incidence of post‐transplantation malignancy is still elusive in Taiwan.

**Method:**

We conducted a nationwide population‐based study. Patients who did not have malignancy history and received kidney transplantation between 2000 and 2010 were enrolled. Specific immunosuppressive users are defined as sustained use (more than 12 months) after renal transplantation. The primary outcome is the development of cancer after kidney transplantation. A Cox proportional hazards model was used to determine the risk of cancer development.

**Result:**

Among 4438 recipients, 559 of them were diagnosed with malignancy after 1 year of transplantation. A total of 742 of recipients were as user of mechanistic target of rapamycin (mTOR) inhibitors. The mTOR users had higher rate of receiving pulse therapy. The hazard ratios (HR) for mTOR inhibitor users with exposure more than 5 years for overall malignancy and urothelial malignancy were 0.68 (95% CI: 0.48‐0.95, *P* = 0.02) and 0.60 (95% CI: 0.36‐0.99, *P* = 0.02), respectively. For the overall mortality and reentry of dialysis, the probability of both groups was similar (overall mortality: *P* = 0.53; reentry of dialysis: *P* = 0.77).

**Conclusion:**

Among the recipients of renal transplantation in Taiwan, mTOR inhibitors with exposure more than 5 years provided a protective role in reducing the risk of overall neoplasm and urothelial malignancy. The probability of reentry of dialysis and overall mortality was similar between the mTORi users and nonusers.

## 1 INTRODUCTION

Renal transplantation has been a part of renal replacement therapy in end‐stage renal disease (ESRD) patients. Because of the improvement in surgical techniques and the advance in immunosuppressive agents, the graft survival and overall survival in the recipients improved in decades.^1^ Although the sources of donors are not sufficient, the percentage of renal transplantation as renal replacement therapy increased gradually. Because of the improvement in the survival, the occurrence of chronic complications such as chronic rejection, interstitial fibrosis, metabolic complications by immuosuppressive agents, or post‐transplantation malignancy increased.^2^, ^3^ Such complications influenced the quality of life and posted threat to the recipients.

Among the post‐transplantation complications, post‐transplantation malignancy is an important complication influencing the graft survival and overall survival.^4^ Among these risk factors, the dysfunction of immune surveillance by immunosuppressive agents is proposed as a contributing factor.^5^ Calcineurin inhibitor‐based regimen is the mainstream immunosuppressive agent in renal transplantation.^6^, ^7^ The immunosuppressive agents abated immune surveillance by regular T cells, and the cytotoxic effect for premalignant cells has been abated after immunosuppressive agents. Besides, it facilitates the action of oncogenic virus and promotes the development of the malignancy.^8^ Calcineurin inhibitors (CNI), impairing the nucleotide excision repair and activation of tumor suppression gene, are commonly mentioned as an initiator for tumor in epidemiologic or in vitro studies.^9^, ^10^ The prevalent post‐transplantation malignancy of specific organ also varies because of the different environmental exposure. In Taiwan, a nationwide population‐based cohort study has been conducted recently for the investigation of the post‐transplantation malignancy in renal transplantation. The most common post‐transplant malignancy in Taiwan was urinary tract and kidney malignancy.^11^, ^12^ As the malignancy occurs, the immunosuppressive agent would be modified and influence the graft survival.^13^, ^14^ Therefore, it posted a great threat to the recipients.

Rapamycin signal network regulates the mRNA translation and cell growth‐related responses. After activating the intracellular phosphatidylinositol4,5‐bisphosphate (PIP2) to phosphatidylinositol 3,4,5‐trisphosphate (PIP3), the downstream Akt kinase activates the mechanistic target of rapamycin (mTOR) complex and induces the cell differentiation and proliferation.^15^ As mTOR complex is important for the T‐cell differentiation and development within thymus, mTOR inhibitor acts as an immune modulator, and its inhibition on Foxp3 +  regulatory cells facilitate the graft survival.^16^ Because it governs the cell proliferation and survival, the genetic alternation or mutation of mTOR signaling is a mechanism of carcinogenesis 17 for treating metastatic renal cell carcinoma, breast cancer, or other hematopoietic malignancy.^18^, ^19^, ^20^ In comparison with CNI, mTOR inhibitors provide less incidence of direct nephrotoxicity, and therefore, early withdrawal of CNI has been a choice in renal transplantation recipients.^4^ Mathews et al^21^ reported that after early withdrawal of cyclosporin (CsA), mTOR inhibitors lowered the incidence of skin cancer as a maintenance therapy after transplantation. Other clinical trials also provided the evidence that mTOR inhibitors use in combination of low‐dose CsA or as alternative of CsA providing the incidence of skin cancer.^22^, ^23^ From the perspectives above, mTOR inhibitor provided a protective role in lessening the post‐transplantation malignancy.

There are reports in the postrenal transplantation malignancy in Taiwan, especially on the analysis of risk factors and incidence of specific solid malignancy.^11^, ^12^ The effect of mTOR inhibitors on postrenal transplantation malignancy is still elusive in Taiwan, although there was evidence of combination of low‐dose mTOR inhibitors with CNI for avoiding post‐transplantation malignancy.^24^ Therefore, the aim of this study is to answer whether the immunosuppressive agent, especially mTOR inhibitor, plays a role in the occurrence of the post‐transplantation malignancy.

## 2 METHOD

### 2.1 Database

We used the inpatient database from the National Health Insurance Research Database (NHIRD). The NHIRD, which was established by the Taiwan Bureau of National Health Insurance (TBNHI) from the National Health Insurance Program, covers more than 99% of Taiwan residents. This database contains insurance information and medical claims of all 23 million insured individuals in Taiwan registered from 2000 to 2013. Disease diagnosis included details of medical orders, procedures, and medical diagnoses with codes based on the International Classification of Diseases, 9th Revision, Clinical Modification (ICD‐9‐CM) in NHIRD. This study was approved by the Ethical Committee of Cardinal Tien Hospital (CTH‐104‐3‐5‐024) and National Health Research Institutes (EC1031006‐E).

### 2.2 Study subject

Patients with new kidney transplants (ICD‐9‐CM V42.0) between 2000 and 2010 were included and followed up to 2013 in this study. The study is based on the NHIRD since 2000 to 2010. We used International Classification of Diseases—9th Revision as the including criteria for the participants.

### 2.3 Study design

We analyzed the subjects after diagnosed with postrenal transplantation diagnosis code (ICD‐9‐CM v42.0 or 996.81). The subjects under the age of 20 years were excluded. The patients with the previous admission history of malignancy (ICD‐9‐CM 140.xx‐208.xx) were excluded. The patients who were expired within 1 year after transplantation were excluded. The patients diagnosed with malignancy within one year after transplantation (admission with diagnosis with ICD‐9‐CM 140.xx‐208.xx) were also excluded. We analyzed the demographic and clinical characteristics of the patients with malignancy (1 year after transplantation) and without malignancy. After then, we regroup the patients into the mTORi users (continuous use more than 1 year after transplantation surgery performed) and mTORi nonusers (exposure of mTORi <1 year after transplantation surgery performed). We analyzed the hazard ratio of occurrence of malignancy based on the mTORi exposure. In order to validate the effect of mTORi on graft failure and overall survival, we compared the reentry of dialysis and mortality between the mTORi users and nonusers.

### 2.4 Pretransplantation covariate assessment

#### 2.4.1 Tested variables

The tested variables included age received transplantation, gender, comorbidities before transplantation, and the modalities of renal replacement treatment before transplantation (hemodialysis (HD) or peritoneal dialysis (PD)).

#### 2.4.2 Comorbidities before transplantation

It included diabetes (ICD‐ 9‐CM 250.xx), hypertension (ICD‐9‐CM 401.xx‐405.xx), ischemic heart disease (CAD; ICD‐9‐CM 410.xx‐414.xx), cerebrovascular disease (CVD; ICD‐9‐CM 430.xx‐438.xx), hepatitis B viral infection (HBV; ICD‐9‐CM V02.61, 070.20, 070.22, 070.30, and 070.32), and hepatitis C viral infection (HCV; ICD‐9‐CM V02.62, 070.41, 070.44, 040.51, and 070.54).

#### 2.4.3 Modalities of renal replacement therapy before renal transplantation

Both HD and PD are the choices of treatment as renal replacement therapy in ESRD patients in Taiwan. The definition of maintenance therapy as HD is defined as consecutively treated with HD and ICD code 585 for more than 3 months. The definition of maintenance therapy as PD is defined as consecutively treated with PD and ICD code 585 for more than 3 months.

### 2.5 Complication after transplantation

#### 2.5.1 Acute rejection

The definition of acute rejection is based on the record of pulse therapy, antithymocyte globulin**,** rituximab, or plasmapheresis during admission.

#### 2.5.2 Occurrence of malignancy

We defined the post‐transplantation malignancy as in‐hospital main diagnosis with ICD‐9‐CM code 140.xx‐208.xx after 1 year of post‐transplantation status. The subjects without malignancy were defined as without occurrence of malignancy after transplantation.

#### 2.5.3 Reentry of dialysis

The definition of reentry of dialysis is consecutively treated HD or PD for more than 3 months after transplantation.

#### 2.5.4 Mortality

The definition of death was defined as discharge due to inpatient death or withdrawal from the NHI program. The date of death was identified from the discharge date of inpatient death or the date of withdrawal from the NHI program.

#### 2.5.5 The use of immunosuppressant agents

1. Definition of maintenance user of specific immunosuppressive agents: The steady use of CNI, mTOR inhibitors, mycophenolate mofetil, steroid, or azathioprine is defined as continuous use of these drugs for more than 12 months after the ICD code v42.0.^25^ As the user of mTOR inhibitors, the definition of mTORi users is as follows: (a) continuous use more than 1 year and (b) prescribed within 1 year after transplantation performed. Those with the started prescription after the occurrence of malignancy were regarded as nonuser for mTOR inhibitors.
2. The effect of accumulative exposure of mTORi: The effect of the accumulative exposure of specific immunosuppressive agents is defined by the days of prescription. We compared the exposure days in three groups (nonuser, exposure duration 1‐5 years, and exposure more than 5 years 26). The index date for mTORi users was the first day of prescription within 1 year after transplantation performed. The patients who discontinued the exposure of mTORi within 1 year were regarded as the mTORi nonusers. In the patients who discontinued the medication within 1 year (12 months), we allocate the patients to the nonusers. Based on the evidences with follow‐up duration more than 5 years, we group the patients with continuous use for 1‐5 years, more than 5 years. The discontinuation of mTORi during 1‐5 years is grouped in the patients with exposure 1‐5 years.

### 2.6 Statistics

All of the data were analyzed by the SAS software, version 9.4 (SAS Institute, Cary, NC, USA), and R software, version 3.4.0. Descriptive data are presented as counts and percentages. Chi‐square test and independent *t* test were used to assess differences in age, gender, comorbidities, and modalities of renal replacement therapy before transplantation and immunosuppressive agents. We conducted modified Cox proportional hazards models to derive hazard ratios (HRs) and 95% confidence intervals (CIs) in relation to the occurrence of malignancy. The survival analysis and the reentry of dialysis between the users and nonusers of mTORi were conducted by Kaplan‐Meier method and compared by log‐rank test. A 2‐tailed *P* value <0.05 indicated a statistical significance.

## 3 RESULT

**Figure 1:**
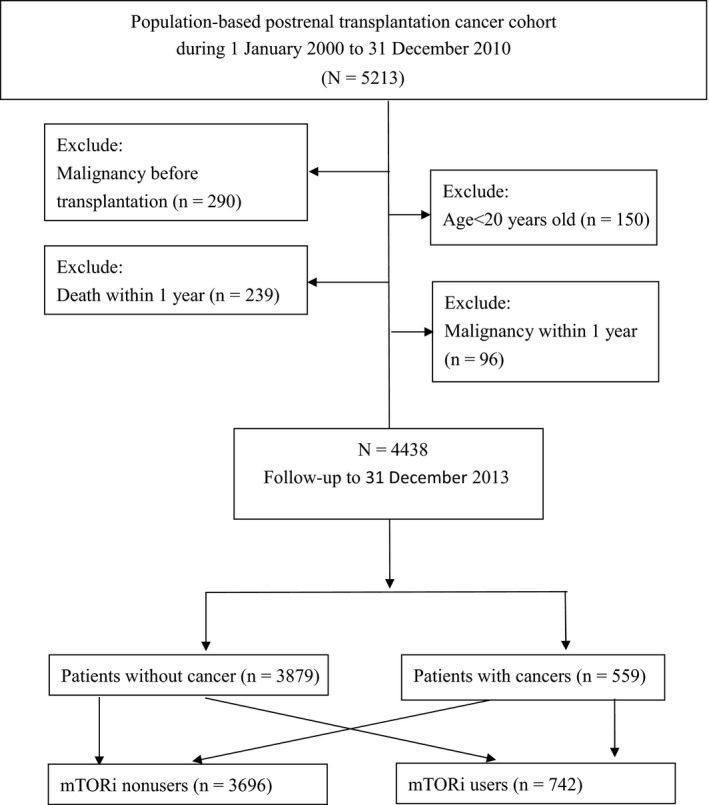
Flowchart for the Patients

Figure 1 demonstrates the flowchart of the study. During 1 January 2000 to 31 December 2010, 5213 patients received renal transplantation with ICD‐9‐CM V42.0. A total of 290 patients were excluded with diagnosis of malignancy before transplantation. The patients younger than 20 years of age were excluded (n = 150). The patients died (n = 239) or diagnosed with malignancy within 1 year (ICD codes 140.xx‐208.xx, n = 96) were excluded. After exclusion, a total of 4438 patients receiving renal transplantation were eligible during the 12‐year dataset period. Among the participants, the number of mTOR inhibitors was 742, and the number of mTOR inhibitor nonusers was 3696.

Table 1 demonstrates the demographic and clinical characteristics of the patients with (cancer) and without (noncancer) malignancy after transplantation. A total of 559 patients were diagnosed after the 1 year of transplantation (12.60% of total subjects). In the cancer group, 64.58% of the patients received transplantation at the age between 45 and 64 years, which was higher than that in the noncancer group (50.76%, *P* < 0.001). Among the noncancer groups, the percentage of patients receiving transplantation with the age 25‐44 years was 45.02%, which was higher than that of the cancer group (29.16%, *P* < 0.001). The percentage of females in the cancer group was 54.74%, which was higher than that in the noncancer group (46.33%, *P* < 0.001). There was no statistical difference in the comorbidities and modality for renal replacement therapy before transplantation. Table 2 lists the demographic and clinical characteristics of the patients as mTORi users or mTORi nonusers. A total of 742 (16.72%) were defined as mTORi users. There was no difference in age between two groups (*P* = 0.18). Among the comorbidities before transplantation, the percentage of hypertension (100% vs 76.89% in nonuser), diabetes mellitus (54.04% vs 19.96% in nonuser), ischemic heart disease (65.90% vs 24.32% in nonuser), HBV infection (17.52% vs 6.68% in nonuser), HCV infection (15.63% vs 5.84% in nonuser), and cerebrovascular disease (14.56% vs 5.25% in nonuser) was all higher in the mTORi user group (*P* < 0.001). The percentage of both HD and PD was higher in the mTOR user group (HD: 82.75% vs 52.16% in nonuser, *P* < 0.001; PD: 30.46% vs18.99% in nonuser, *P* < 0.001). In the mTOR users, 90 patients were diagnosed with cancer (12.13%). The average following‐up years for mTORi nonusers, mTORi users with exposure between 1‐5 years and users with exposure more than 5 years were 8.14 ± 3.31 years, 8.07 ± 3.37 years and 7.83 ± 3.34 years respectively. In the nonusers, 702 patients were diagnosed with cancer (12.64%). There was no statistical difference in the percentage of overall malignancy between mTORi used and nonusers (*P* = 0.67). The commonest types of malignancy were urothelial (n = 259, 46.33%), kidney (n = 70, 12.52 %), liver (n = 79, 14.13 %) and digestive system (n = 59, 10.55 %). There was no statistical difference in the percentage of subgroup malignancy between two groups. There was no statistical difference in the percentage of overall mortality during the follow‐up duration (*P* = 0.51).

**Table 1:** Demographic and clinical characteristics of the patients with and without cancer

	Noncancer		Cancer		*P* value
	N	%	N	%	
Total subjects	3879	87.40	559	12.60	
The age receiving transplantation (y)					
20‐44	1747	45.04	163	29.16	<0.0001
45‐64	1969	50.76	361	64.58	
65+	163	4.20	35	6.26	
Sex					
Female	1797	46.33	306	54.74	0.00
Male	2080	53.62	252	45.08	
Comorbid disorders					
Diabetes mellitus	964	24.85	145	25.94	0.58
Hypertension	3134	80.79	450	80.50	0.87
Ischemic heart disease	1213	31.27	175	31.31	0.99
Hepatitis B	334	8.61	43	7.69	0.47
Hepatitis C	297	7.66	35	6.26	0.24
Cerebrovascular disease	269	6.93	33	5.90	0.37
Modality of Renal Replacement therapy before transplantation					
Hemodialysis	2213	57.05	329	58.86	0.42
Peritoneal dialysis	820	21.14	108	19.32	0.32
Using mTORi duration					
Never used	2990	77.08	430	76.92	0.93
Within 1 y	238	6.14	38	6.80	
Within 1‐5 y	390	10.05	55	9.84	
Over 5 y	261	6.73	36	6.44	

**Table 2:** Demographic and clinical characteristics of mTORi users and nonusers

	mTORi nonusers^a^		mTORi users		*P* value
	N	%	N	%	
Total subjects	3696	83.28	742	16.72	
Age group (y)					
20‐44	1599	43.26	311	41.91	0.18
45‐64	1924	52.06	406	54.72	
65+	173	4.68	25	3.37	
Sex					
Female	1765	47.75	338	45.55	0.28
Male	1929	52.19	403	54.31	
Comorbid disorders					
Diabetes mellitus	708	19.16	401	54.04	<0.0001
Hypertension	2842	76.89	742	100.00	<0.0001
Ischemic heart disease	899	24.32	489	65.90	<0.0001
Hepatitis B	247	6.68	130	17.52	<0.0001
Hepatitis C	216	5.84	116	15.63	<0.0001
Cerebrovascular disease	194	5.25	108	14.56	<0.0001
Modalities of renal replacement therapy before transplantation					
Hemodialysis	1928	52.16	614	82.75	<0.0001
Peritoneal dialysis	702	18.99	226	30.46	<0.0001
Cancer diagnosed	467	12.64	90	12.13	0.35
Within 1‐3 y	146	31.26	22	24.44	
Within 3‐5 y	117	25.05	22	24.44	
Over 5 y	204	43.68	46	51.11	
Mortality	517	13.99	97	13.07	0.51
Overall cancer	469	12.69	90	12.13	0.67
Urothelial malignancy	219	5.93	40	5.39	0.57
Kidney malignancy	58	1.57	12	1.62	0.92
Liver malignancy	66	1.79	13	1.75	0.95
Digestive system malignancy	48	1.30	11	1.48	0.69

a Nonusers: Included subjects who never used or using <1 y.

Table 3 lists the percentage of immunosuppressive agents used in the two groups. The mTORi users had more experience in acute rejection (16.04% vs 10/17% in mTORi nonusers, *P* < 0.0001). The patients in mTORi user group also had higher percentage of using MMF (93.30% vs 85.25%, *P* < 0.0001), azathioprine (8.49% vs 5.38%, *P* < 0.0001), steroid (96.23% vs 92.97%, *P* < 0.0001), and CNIs (97.04% vs 92.94%, *P* < 0.001) as the maintenance immunosuppressive agents.

**Table 3:** The use of immunosuppressive agents between the mTORi users and nonusers

	mTORi nonusers^a^		mTORi users		*P* value
	N	%	N	%	
Immunosuppressive agents	3696		742		
Transplant rejection	376	10.17	119	16.04	<0.0001
Calcineurin inhibitor	3435	92.94	720	97.04	<0.0001
MMF	3151	85.25	696	93.80	<0.0001
Azathioprine	199	5.38	63	8.49	0.001
Steroid	3436	92.97	714	96.23	0.001

a Nonusers: Included subjects who never used or using <1 y.

Table 4 demonstrates propensity score matching for the occurrence of post‐transplantation malignancy. In the propensity score matching for sex, gender, comorbidities, modalities before transplantation, and immunosuppressants used, mTORi did not provide a protective effect for overall malignancy or other subgroups of malignancy. Table 5 demonstrates multivariable Cox regression for the hazard ratio of the occurrence of post‐transplantation malignancy based on the days of exposure of mTORi in the total subjects after adjustment for age, gender, comorbidities, and modalities of renal replacement therapy before transplantation and immunosuppressive agent. The patients with exposure more than 5 years had a protective effect on the occurrence of overall malignancy (HR: 0.68, 95% CI: 0.48‐0.95, *P* = 0.02) and urothelial malignancy (HR: 0.60, 95% CI: 0.36‐0.99, *P* = 0.02). The adjustment process was demonstrated in the supplement data (Table S1).

**Table 4:** Propensity score matching of mTORi use for the occurrence of post‐transplantation malignancy

mTORi	All cancer		Urothelial malignancy		Kidney malignancy		Liver malignancy		Digestive system malignancy	
	Hazard ratio	95% CI	Hazard ratio	95% CI	Hazard ratio	95% CI	Hazard ratio	95% CI	Hazard ratio	95% CI
Never used	1.00		1.00		1.00		1.00		1.00	
Users	0.67	0.44, 1.03	0.66	0.35, 1.26	0.49	0.16, 1.51	0.91	0.31, 2.67	0.88	0.25, 3.15
Never used	1.00		1.00		1.00		1.00		1.00	
Using 1‐5 y	0.88	0.65, 1.19	0.80	0.51, 1.24	0.52	0.21, 1.31	1.58	0.78, 3.17	0.71	0.28, 1.81
Using more than 5 y	0.68	0.48, 0.95	0.60	0.36, 0.99	0.53	0.20, 1.42	0.51	0.18, 1.45	0.80	0.30, 2.11

Adjusted for age, gender, comorbidities, and modalities of renal replacement therapy before transplantation and immunosuppressive agent.

**Table 5:** Adjusted hazard ratio for the occurrence of overall malignancy and subgroups of malignancy based on the exposure of mTORi

mTORi	All cancer		Urothelial malignancy		Kidney malignancy		Liver malignancy		Digestive system malignancy	
	Hazard ratio	95% CI	Hazard ratio	95% CI	Hazard ratio	95% CI	Hazard ratio	95% CI	Hazard ratio	95% CI
Never used	1.00		1.00		1.00		1.00		1.00	
Users	0.67	0.44, 1.03	0.66	0.35, 1.26	0.49	0.16, 1.51	0.91	0.31, 2.67	0.88	0.25, 3.15
Never used	1.00		1.00		1.00		1.00		1.00	
Using 1‐5 y	0.88	0.65, 1.19	0.80	0.51, 1.24	0.52	0.21, 1.31	1.58	0.78, 3.17	0.71	0.28, 1.81
Using more than 5 y	0.68	0.48, 0.95	0.60	0.36, 0.99	0.53	0.20, 1.42	0.51	0.18, 1.45	0.80	0.30, 2.11

**Figure 2:**
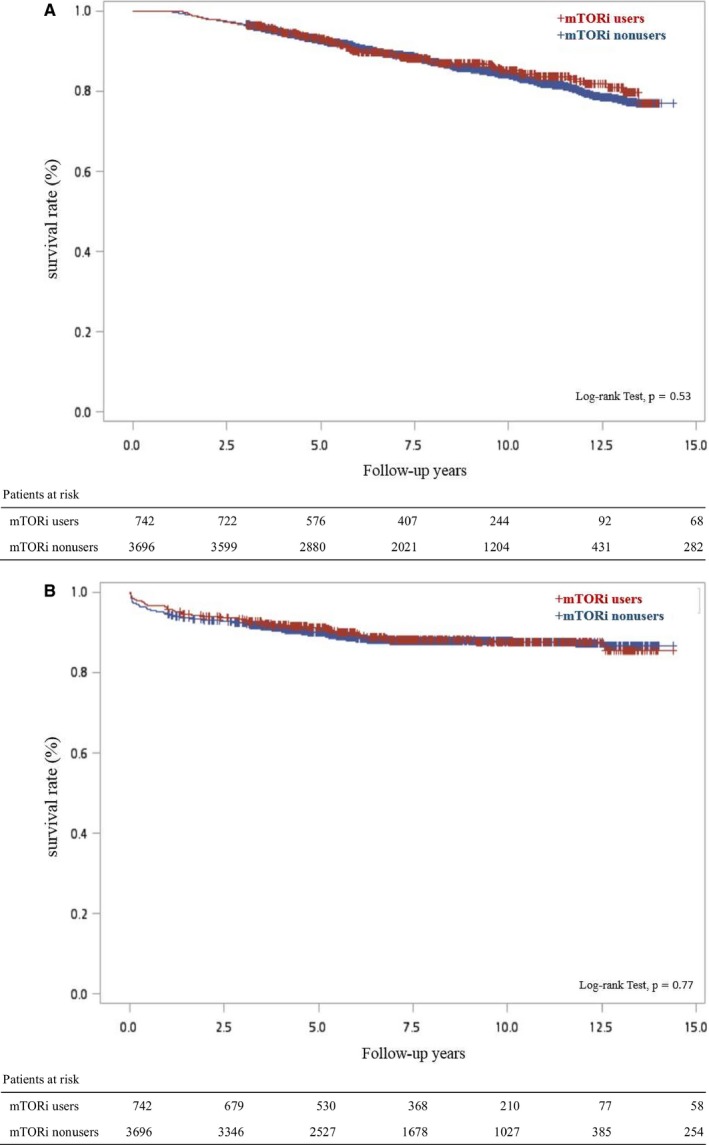
Kaplan‐Meier Curve for the Overall Survival (A) and Reentry for Dialysis (B) Between the mTORi Users and Nonusers

Figure 2 demonstrates the effect of mTORi exposure on the mortality and reentry of dialysis by the Kaplan‐Meier plot. For the overall mortality and reentry of dialysis, the probability of both groups was similar (overall mortality: *P* = 0.53; reentry of dialysis: *P* = 0.77).

## 4 DISCUSSION

In this cohort study, we reviewed the incidence of post‐transplantation malignancy of the recipients and the risk factors for the occurrence in Taiwan. We also analyzed the clinical characteristics of the mTORi uses after transplantation and the effect of mTORi exposure on the occurrence of post‐transplantation malignancy. The most common malignancy was urothelial malignancy. In the mTORi users, the percentage of pretransplantation comorbidities was higher, but the percentage of overall malignancy was similar to the nonusers. After adjusting for the age, gender, comorbidities, and modalities of pretransplantation renal replacement therapy, mTORi exposure more than 5 years had protective effect on the overall and urothelial malignancy.

In previous studies, it has been noticed that the urothelial malignancy in postrenal transplantation was the most common malignancy in Taiwan, and the pretransplantation renal placement therapy with HD was predictive of the urothelial malignancy.^11^, ^12^ Our study provides the same results. Chien et al^27^ reported that among female HD patients in Taiwan, urothelial cancer was the most common malignancy. According to the epidemiologic studies in Taiwan, Chinese herb use was a risk factor for the occurrence of the urothelial malignancy and an important contributor for the ESRD.^28^, ^29^ The immune dysfunction status such as diabetes mellitus or uremic milieu was a contributing factor for the malignancy in ESRD.^30^ However, it is still inconclusive whether uremic milieu induces premalignant transformation of urothelial epithelial cells. Indoxyl sulfate has been noticed to suppress the multidrug resistance protein 4 and breast cancer resistance protein in vitro.^31^ Besides, it has been noticed that indoxyl sulfate may induce proximal tubule senescence by activating p53 gene.^32^ The surveillance for premalignant transformation was difficult in dialysis patients because observation for hematuria would be lessened by relative insufficient residual renal function.^33^ Therefore, it is difficult to validate the nature history of urinary malignancy in ESRD patients in Taiwan.^29^ Further investigation on the surveillance of early malignant change in HD should be emphasized.

Our studies revealed that at least 5 year of mTOR inhibitor exposure after the renal transplantation was protective in urothelial and overall malignancy occurrence. Several studies also provide the evidences of the protective effect on lessening the post‐transplantation malignancy. From the collaborative transplant study data, the use of mTOR inhibitors as the immunosuppressive agents lessened the incidence of basal cell carcinoma after transplantation.^34^ Among patients with nonmelanoma skin cancer history, mTOR inhibitors lessened the incidence of postrenal transplant skin cancer rather than CNI.^35^ At the same time, more than 12 months of use of mTOR inhibitors also lowered the incidence of nonmelanoma skin cancer in recipients previously treated with CNI.^25^ The cohort studies based on the national registry database provided a clue that mTOR inhibitors provide a protective role in treating the most common malignancy after transplantation. Among urothelial carcinoma, *PTEN‐PI3K‐AKT* pathway was important in tumorigenesis.^36^ In human urothelial carcinoma, higher Akt and *β‐*catenin expressions were associated with higher invasiveness in urothelial cancer cells, and the deletion or mutation of p53 gene and phosphatase and tensin homolog (PTEN) activates the Akt and further tumorigenesis.^37^ Wu et al^36^ also provided the in vivo evidence that mTOR Rictor‐dependent Akt activation was an important pathway for urothelial carcinoma, and such activation could be inhibited by rapamycin. Although mTOR inhibitors have not been applied as the first‐line treatment for treating invasive or metastatic urinary bladder cancer, inhibition on mTOR and its downstream signal has been applied in vitro and in clinical trials. In postrenal transplantation status, polyomavirus replication was predictive of bladder cancer development.^38^ Yen et al^39^ also noticed that the use of mTOR inhibitor decreases the polyomavirus viral loading in comparison with other immunosuppressive agents. Previous studies in Taiwan did not show that the maintenance use of mTOR inhibitor provided a protective role in urothelial malignancy after transplantation.^40^ Kao et al defined the mTORi users as having exposure more than 30 days instead. However, the effect may not be easily demonstrated due to the limited days of exposure. The clinical trials involving the mTORi in renal transplantation would adopt duration of more than 1 month. Based on the literature reviews, we adopted the definition of mTORi exposure as exposure more than 1 year. We defined the mTORi users as those who received the treatment for the first time within 1 year after transplantation had been performed. We use such definition to avoid the adjustment of medication due to metabolic complications induced by other immunosuppressant agents. Lebrachu et al^26^ provided the evidence that the users with sustained mTORi exposure more than 5 years had better estimated glomerular filtration rate than CNI users. The ZEUS study provided the evidence that mTORi‐based regimen was associated with a significant improvement in renal function for at least five years.^41^ Therefore, we divided patients into sustained exposure more or <5 years. Our results revealed that the sustained use of mTORi more than 5 years was protective for malignancy occurrence, which was consistent with the result of Lebrachu et al's report. According to the self‐report study, the adherence of immunosuppressant agents was high in the kidney transplantation recipients in Taiwan 42 even though the longer post‐transplantation duration was negatively related to the adherence. Therefore, to investigate the factors enhancing adherence in mTORi might help to prevent the occurrence of post‐transplantation malignancy. Further studies might be needed.

It is interesting that the percentage of comorbidities such as hypertension, diabetes mellitus, ischemic heart disease, and cerebrovascular disease was higher in the mTORi users. In contrast to CNI, the nephrotoxicity and hypertension were less common in the mTORi users,^4^ and pretransplantation comorbidities might influence the clinicians’ decision in choosing the immunosuppressive agents. Hyperglycemia and hyperlipidemia were linked to the mTOR inhibitors, but Lamming et al^43^ reported that mTORi provided longevity beyond the insulin resistance. Insulin resistance is associated with the inhibition of mechanistic target of rapamycin complex 1, and the inhibition of mTORC2 lowers hepatic gluconeogenesis. Besides, switching to mTORi from CNI provided better glycemic control in selected patients.^44^ These findings might explain why the mTORi users in our study group had similar overall survival to the mTORi nonusers. In our database, the percentage of acute rejection was higher in the mTORi users. Although mTOR inhibitors provided antineoplastic effect in preventing malignancy, it has been noticed that the avoidance of CNI increased the rate of rejection and graft dysfunction.^4^ The chronic antibody‐mediated rejection after sustained exposure to mTORi might influence the graft survival.^45^ Recent meta‐analysis and literature reviews provided the evidence that early withdrawal of CNI and introduction of mTORi might induce the de novo glomerulonephritis and graft loss.^46^, ^47^ However, the Cochrane database reported that mTORi might increase the acute rejection, but the effect on long‐term graft loss was still uncertain.^4^ From our result, the percentage of reentry of dialysis in the mTORi users is similar to the patients with mTORi nonusers. To sum up the results above, the regimen with mTORi is not inferior with the mTORi nonusers, but further clinical trials are needed to validate the benefits of mTOR inhibitor in Taiwanese renal transplantation.

There were several limitations in our study. First, like all registry studies, our analysis has the limitation that the accuracy of reporting is not equivalent to that in a prospective trial. Because the registry system could not provide the definite pathology of specific cancer and cannot present the staging of each individual, we could not analyze the role of immunosuppressive agents in different stages of malignancy. Further validation should be performed thereafter. Besides, the effect of other immunosuppressive agents provided a neutral effect on the occurrence of postrenal transplantation malignancy. Although clinical trials provided the safety and efficacy of monotherapy with mTOR inhibitor after transplantation,^48^ most patients in Taiwan received combination therapy as maintenance therapy. Further studies on the effect of combination therapy may be considered to find out the optimal regimen in transplantation recipients. Moreover, our database could not provide the accumulative number of tables. The database could provide the exposure duration instead.

In conclusion, from the nationwide cohort study of kidney transplantation in Taiwan, we found that mTOR inhibitors with exposure more than 5 years provided a protective role in reducing the risk of overall neoplasm and urothelial malignancy. The probability of reentry of dialysis and overall mortality was similar between the mTORi users and nonusers.

## ETHICS

This study was approved by the Ethical Committee of Cardinal Tien Hospital (CTH‐104‐3‐5‐024) and the National Health Research Institutes (EC1031006‐E).

## DISCLOSURE

The results of this study were presented as an abstract at Asian Transplantation Week 2017.

## CONFLICT OF INTEREST

The authors declare no conflict of interest.
